# Comprehensive measures succeeded in improving early detection of leprosy cases in post-elimination era: Experience from Shandong province, China

**DOI:** 10.1371/journal.pntd.0007891

**Published:** 2020-02-20

**Authors:** Tongsheng Chu, Dianchang Liu, Pengcheng Huai, Xinlong Chen, Shenghui Han, Shumin Chen, Furen Zhang

**Affiliations:** 1 Shandong Provincial Hospital for Skin Disease, Shandong University, Jinan, Shandong, China; 2 Shandong Provincial Institute of Dermatology and Venereology, Shandong Academy of Medical Sciences, Jinan, Shandong, China; 3 Jining Hospital for Skin Disease Control and Prevention, Jining, Shandong, China; Swiss Tropical and Public Health Institute, SWITZERLAND

## Abstract

**Background:**

A few new leprosy cases still can be seen in Shandong province after elimination. In post-elimination era, government commitments dwindled and active case-finding activities were seldom done. Most of the cases were detected by passive modes and advanced cases with longer delay and visible disability were common.

**Materials and methods:**

Comprehensive measures including health promotion, personnel training, reward-offering, symptom surveillance and a powerful referral center were implemented in the past decade. The diagnosis of leprosy was mainly based on three cardinal clinical signs. Two-group classification system developed by the WHO was used and cases were classified into multibacillary (MB) type or paucibacillary (PB) type. Cases detected during period 2007–2017 were analyzed and associated factors of grade 2 disability (G2D) were explored.

**Results:**

231 new leprosy cases detected during 2007–2017 were analyzed. The mean age at diagnosis is 51.7±16.0 years and the number of males, peasants, illiterates, MB cases, G2D cases and immigrants were 130(56.3%), 221(95.7%), 73(31.6%), 184(79.7%), 92(39.8%) and 40(17.3%) respectively. 181(78.4%) cases were reported by skin clinics and 152 (65.8%) cases came from formerly high endemic counties/districts. The annual number of new cases showed a decreasing trend, from 42 cases in 2008 to 13 cases in 2017. 92 (39.8%) cases presented with G2D at diagnosis. The annual proportion of new cases with G2D declined from 50% in 2008 to 23% in 2017. PB type (OR = 2.76, 95% CI, 1.43–5.32), >12 months of patient delay (OR = 2.40, 95% CI, 1.38–4.19), >24 months of total delay (OR = 4.35, 95% CI, 2.33–8.11), detected by non skin-clinic (OR = 3.21, 95% CI, 1.68–6.14), known infectious source (OR = 1.77, 95% CI, 1.01–3.12) were associated with G2D.

**Conclusion:**

A few scattered cases still can be seen in post-elimination era and some kind of leprosy control program is still necessary. Government commitments including adequate financial security and strong policy support are vital. Comprehensive case-finding measures including health promotion, personnel training, reward-offering, with an emphasis on former high or middle endemic areas, are necessary to improve early presentation of suspected cases and to increase suspicion and encourage participation of all relevant medical staff. Symptom surveillance based on a powerful transfer center may play an important role in the early detection of new cases in post-elimination era.

## Author summary

After elimination, most of the leprosy cases were detected passively and advanced cases with longer delay and visible disability were common. In the past decade, comprehensive case-finding measures including health promotion, personnel training, reward-offering symptom surveillance and a powerful referral center were implemented successively in Shandong province after the restoration of government commitments. 231 cases detected during 2007–2017 were analyzed and factors associated with G2D were explored. Annual proportion of G2D among new cases showed a declining trend, from 50% in 2008 to 23% in 2017. PB type, longer delay in diagnosis, detected by other than skin-clinics and known infectious sources were associated with G2D. Even in late phase of leprosy control, some kind of leprosy control program is still necessary. Comprehensive case-finding measures based on government commitments would be beneficial to improve early detection of leprosy cases.

## Introduction

Leprosy, or Hansen`s disease, caused by *mycobacterium leprae*, is a chronic infectious disease with a long incubation [1] and preferably affects skin and peripheral nerve systems [2]. If not treated timely, leprosy can result in permanent damage to peripheral nerves and may lead to amputations and disabilities [3]. Leprosy is considered as a severe public health issue mainly because of its potential disabling characteristics [4]and relevant social, economic consequences.

Since the successful introduction of multidrug therapy (MDT) recommended by the World Health Orgnization (WHO) in 1981, the global prevalence rate of leprosy declined dramatically from about 12 million in 1985 [5] to 171,948 in 2016[6]. The WHO elimination goal, defined as less than 1 per 10,000, was achieved in 2000 at global level [7]. At the beginning of 2007, only 4 countries failed to achieve the goal of elimination of leprosy [8]. Despite of this great progress, the global number of new cases was almost static in the past decade [9]. In 2016, there were 214,783 newly detected cases globally, which showed a marginal increase from 211,973 in 2015 [6].

Delay in diagnosis augments the transmission of infection and may lead to progression of the disease, irreversible disabilities [10, 11]. The importance of early diagnosis of leprosy can never be overemphasized in terms of reducing the reservoirs of bacilli and decreasing the risks of deformities [12]. For long time, the proportion of grade 2 disability (G2D), defined as any visible disabilities such as ulcers, claw fingers/toes and muscle wasting [13], among newly detected cases had been set as one of the most important epidemiological indicators to evaluate case-finding activities among different leprosy control programs [12].The global number of new cases with G2D, however, showed no decreasing trend from 12392 in 2006[14] to 12,437 in 2016 [6], indicating a failure in early detection. In April 2016, WHO launched a 5-year global leprosy strategy and reduction in the rate of new G2D cases was set as one of the three main targets at global level [15]. This calls for increased efforts to infuse new vigor into leprosy control program to detect and treat leprosy cases early. In the past several decades, significant delays with wide variations in diagnosis of leprosy had been reported in several countries [10,11,16] and possible reasons had also been identified to promote early diagnosis. However, there is little literature on the measures taken and evaluation of relevant effectiveness on reduction of proportion of G2D cases among newly detected leprosy patients.

Shandong province, a semi-island, is located in the eastern part of China. This coastal province is divided into 16 municipalities and subdivided into 137 counties/districts. Shandong Provincial Institute of Dermatology and Venerology (SDPIDV) is responsible for leprosy control of the whole province. With a population of some 100 million and a land area of 153 thousand square kilometers, Shandong is one of the most densely populated provinces in China. With more than 54 thousand of cases had been registered since the establishment of leprosy control program in 1950s, Shandong was one of the most leprosy-endemic provinces in China. MDT recommended by WHO was implemented in 1986 and elimination goal of leprosy by Chinese level, defined as less than 1 per 100,000, had been achieved in 1994.

The celebration of the leprosy progress resulted in loss of political commitment [17]. During the last two decades, support for vertical leprosy program gradually reduced [18] and some vertical leprosy control programs were integrated into general health services. Active case-finding activities, such as population survey and rapid village survey, were seldom carried out for cost-benefit reasons and nearly 80% of the leprosy cases were detected by passive case-finding modes including skin-clinics, reported by others, referrals, etc. In the subsequent several years after elimination, the prevalence rate of leprosy in some former high-endemic counties exceeded the standard again.

In 2008, shortly after the World Leprosy Day, Shandong provincial special fund for leprosy was restored under the proposal of a provincial governor. Since then, comprehensive measures focused on early detection of leprosy cases have been performed in this province. To verify the role of comprehensive measures in early detection of leprosy cases, the trends in proportion of G2D patients among newly notified cases during 2007–2017 were explored and factors associated with G2D were also ascertained.

### Comprehensive measures adopted

In the past decade, the following comprehensive measures, most of which included in *Shandong provincial program of eliminating leprosy hazard (2011–2020)*, were implemented in Shandong province:

#### 1) Health promotion campaigns

A poster, a leaflet and a 5-minute video in electronic edition, which targeted specifically for general population, were designed by SDPIDV and free to use for all medical institutions. Health promotion campaigns were performed annually, usually combined with routine leprosy control activities and daily medical activities, to enhance awareness of leprosy and promote early presentation and self-reporting of the public.

#### 2) Personnel training

A 30-min powerpoint presentation on leprosy, mainly focused on the common, typical symptoms of leprosy and misdiagnosis experiences encountered by sufferers, was included in all academic activities of 3 provincial associations of leprosy, dermatologists and medical doctors, to improve knowledge and skill in early diagnosis of leprosy among dermatologists. Approximately 500 dermatologists were trained annually. Each rural doctor was given a leprosy control manual and trained annually by leprosy control programs at county level.

#### 3) Case report reward

From 2006 on, for each confirmed case of leprosy, a reward of 400 Yuan (59.5 US dollars) was provided for the first people who think of the possibility of leprosy. If a case is reported by a person in the same community, however, the reporter will not get the award for fear of causing stigmas. Case report reward was provided by Shandong provincial government and managed by SDPIDV. In 2013, this reward was increased to 5000 Yuan (744 US dollars) for each case with <12 months of delay in diagnosis and without visible disabilities, and 1000 Yuan (148.6 US dollars) for each of other cases. The reporter can also get a certificate of honor issued by Shandong provincial association of leprosy, which is useful for position promotion.

#### 4) Monitoring of Leprosy symptoms

In recent years, more and more vertical leprosy control programs were integrated into general hospitals or centers of disease control (CDCs) and Concerns arose about a potential decline in the quality of leprosy control, especially for early detection of leprosy cases. In August 2016, Shandong provincial health bureau issued *Implementation Plan of Leprosy Symptom Monitoring in Shandong Province*, which was officially implemented on January 1, 2017. Each of the 107 first-class general hospitals and 55 leprosy control institutes in the province was asked to transfer at least one suspected case of leprosy per month. In each above-mentioned medical unit, dermatologists are responsible for screening suspected leprosy cases reported by other departments and transferring those they believe suspicious to SDPIDV. The performance of referral was included in the assessment indicators of the medical units.

#### 5) A powerful referral center

To diagnose leprosy as accurate as possible, a powerful referral center with experienced staff and robust laboratory is also vital to establish or exclude the diagnosis of leprosy. Leprosy workers in SDPIDV were trained annually at national center of leprosy control and had participated in the diagnosis, treatment and follow-up of all new cases in Shandong province since 2007. In a low endemic situation, this is conducive to maintaining a high level of expertise and management skills for leprosy workers at the provincial level. Pathology department of SDPIDV, founded in 1955, could provide technical support for the diagnosis of leprosy and other skin diseases. Since 2014, Polymerase Chain Reaction (PCR) technology had been introduced, which can further improve the diagnostic accuracy of PB cases, especially those with atypical clinical symptoms, negative smear results and nonspecific pathological changes, and distinguish leprosy from other mycobacterial diseases.

## Materials and methods

### Diagnosis and classification of leprosy

In order to ensure the accuracy of the diagnosis of leprosy, from 2007 on, all the suspected leprosy cases in Shandong province were asked to be transferred to SDPIDV for confirmation. For the seriously disabled and frail elderly, leprologists from SDPIDV would go to local hospital or the suspect`s home for examination and the specimens would be taken back to SDPIDV for inspection. The diagnosis of leprosy is based on three cardinal clinical signs, i.e. anaesthetic skin lesions, enlarged peripheral nerves and acid-fast bacilli in the skin smear. Histopathological examination was also implemented for nearly all the patients. Two-group classification system developed by the WHO was used. Patients with 6 or more skin lesions, or positive bacteriological results (either through skin smear, biopsy or PCR) were classified as MB patients. While the patients with 1–5 skin lesions and negative bacteriological results were classified as PB patients.

### Study population

This SDPIDV based descriptive retrospective study was based on all the newly detected leprosy patients in the period 2007–2017.

### Data collection and statistical analysis

Early in 2007, a new file including demographic information, help-seeking habits and epidemiological data was designed and pretested before use. The average time of data collection for each patient was about 30 minutes. All the data were entered into software Statistical Package for Social Sciences (SPSS) version 22.0. Qualitative variables were presented as frequencies and percentages, while quantitative variables were presented as the mean and the standard deviation. The chi-square test was used to compare categorical variables. Student’s *t* test was used to compare quantitative variables. Logistic regression was performed to explore associated factors of G2D. A *P* value less than 0.05 was considered as significant.

### Ethical statement

This study was approved by SDPIDV ethical committee. An informed consent was signed by literate individuals and fingerprinted by illiterate patients. All data were anonymized and the patients’ confidentiality was strictly respected in the data processing and analysis.

## Results

### Demographic information

During the study period, a total of 232 newly detected leprosy cases were notified. 1 case was excluded from this study due to mental reasons and the data of 231 cases were analyzed finally. Among the 231 patients, there were 130 males and 101 females, with a male to female ratio of 1.30. The mean age at diagnosis was 51.7±16.0 years, ranging from 18 to 85 years. The mean age of male patients (53.2±15.6 years) was slightly older than that of female (49.7±16.4 years), but the difference was not significant (P = 0.098). No child case (less than 15 years old) was notified in this period, although the onset age of 3 cases were <15 years. The majority (221, 95.7%) of the patients was peasants or unskilled laborers, except for 4 workers, 2 teachers, 1 student, 1 long-distance driver and 1 rural doctor.

In terms of educational level, 73(31.6%) were illiterates, 75(32.5%) had primary school education, 83(35.6%) had junior high school or above education. The proportion of illiterate was much higher in female than that of male (48.5% *vs*. 18.5%, *P =* 0.001).

### Epidemiological information

The majority (184, 79.7%) of patients was classified as MB type and 47 (20.3%)cases were classified as PB patients. Among the 231 newly detected cases, 158 (68.4%) cases denied any contact with a leprosy case. 47 (20.3%) cases had a family ground of at least one leprosy case. 26 (11.3%) cases reported contacts with non-relative leprosy patients. The most common detection mode was through skin-clinics (181, 78.4%), followed successively by referral by other departments of general hospitals (13, 5.6%), report by others (12, 5.2%), rapid village survey (11, 4.8%), contact examination (7, 3.0%) and self-report (7, 3.0%).

### Distribution of cases

Historically, Weifang, Linyi and Qingdao are the three most endemic municipalities in Shandong province, with the accumulative number of leprosy cases ranks first, second and third respectively. During period 2007–2017, the 231 newly detected leprosy cases were scattered in 17 municipalities, 94 counties/districts and the distribution was not balanced. At municipal level, there were 46 cases, 40 cases and 23 cases in the above 3 municipalities, respectively. The disease burden of the above 3 municipalities accounted for nearly half of the total case burden. At county level, the disease burden of 32 counties/districts, most of which were formerly endemic areas and each of which had 3 or more cases, accounted for the majority (152/231, 65.8%) of newly detected cases.

Out of the 231 cases, 191 (82.7%) were born in Shandong province and 40 (17.3%) were immigrants. Most of the immigrated cases came from Yunnan province and Sichuan province, both of which are highly endemic provinces in southwestern part of China. Of those 40 immigrants, 31 were female and 9 were male. The proportion of immigrants among female is significantly higher than that of male (*P<0*.*001 OR = 5*.*954*). the mean age of immigrated patients was 39.5±12.2years, which is significantly younger than that of native patients (54.3±15.6 years, *P*<0.001).

### Trends in new case detection

**Fig 1:**
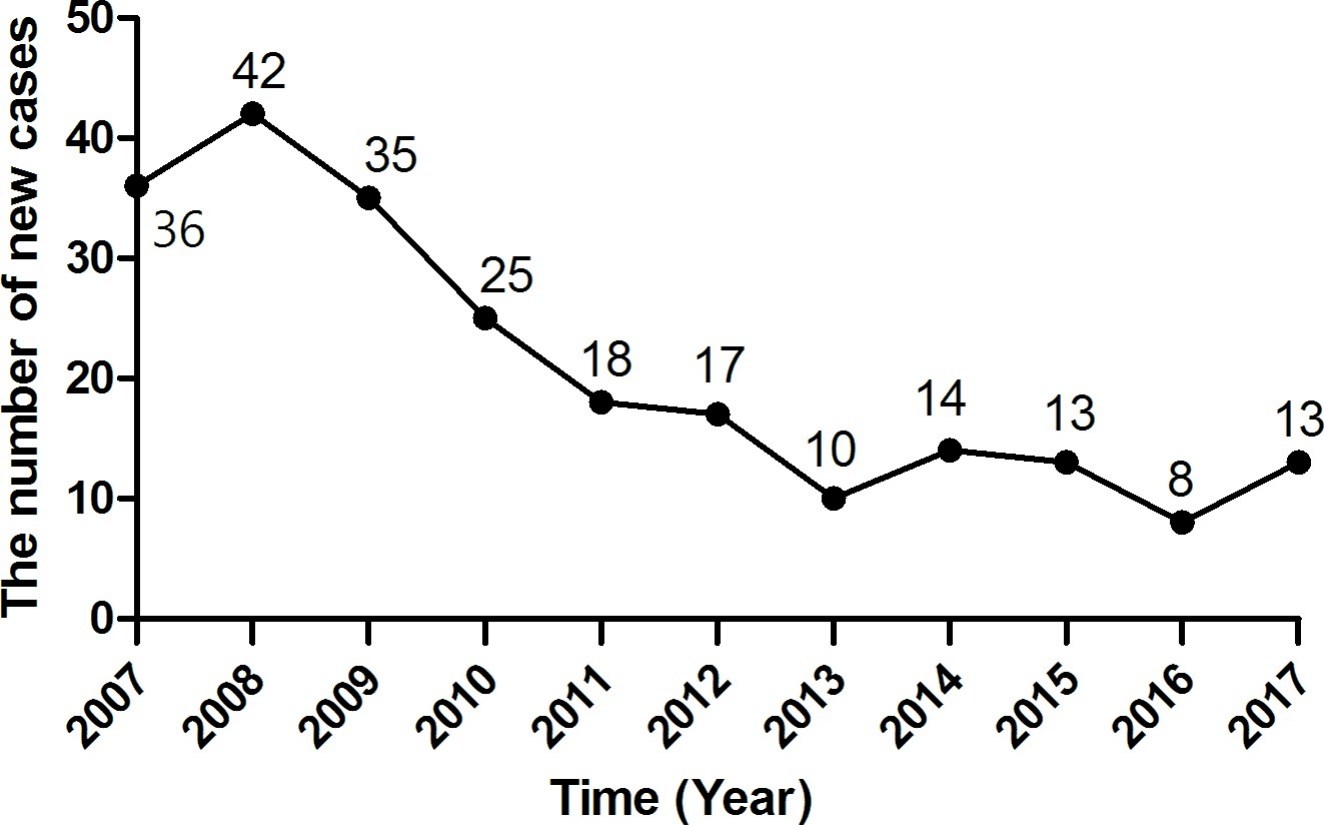
The annual number of newly detected leprosy cases.

The numbers of new cases detected each year showed a decreasing trend from 2007 to 2017 (Fig 1), ranging from 42 in 2008 to 8 in 2016. From 2007 to 2009, the number of annual leprosy cases was rather stagnant. A drastic reduction occurred between 2009 and 2013. From 2014, the annual number of cases reached a lower plateau.

### Delay in diagnosis

The mean interval of patient delay, health system delay and total delay, which defined respectively as the time elapsed between the onset of the disease and first visit to a medical doctor, first visit to a doctor and establishment of diagnosis of leprosy, the onset of the disease and the establishment of diagnosis, were calculated in months. The mean and the median patient delay were 30.1 months and 6 months respectively, ranging from 0 to 420 months. The mean and the median health system delay were 34.3 months and 13 months respectively, ranging from 0 to 373 months. The mean and the median total delay were 64.1 months and 36 months respectively, ranging from 1 to 420 months.

### Grade 2 disability (G2D)

**Fig 2:**
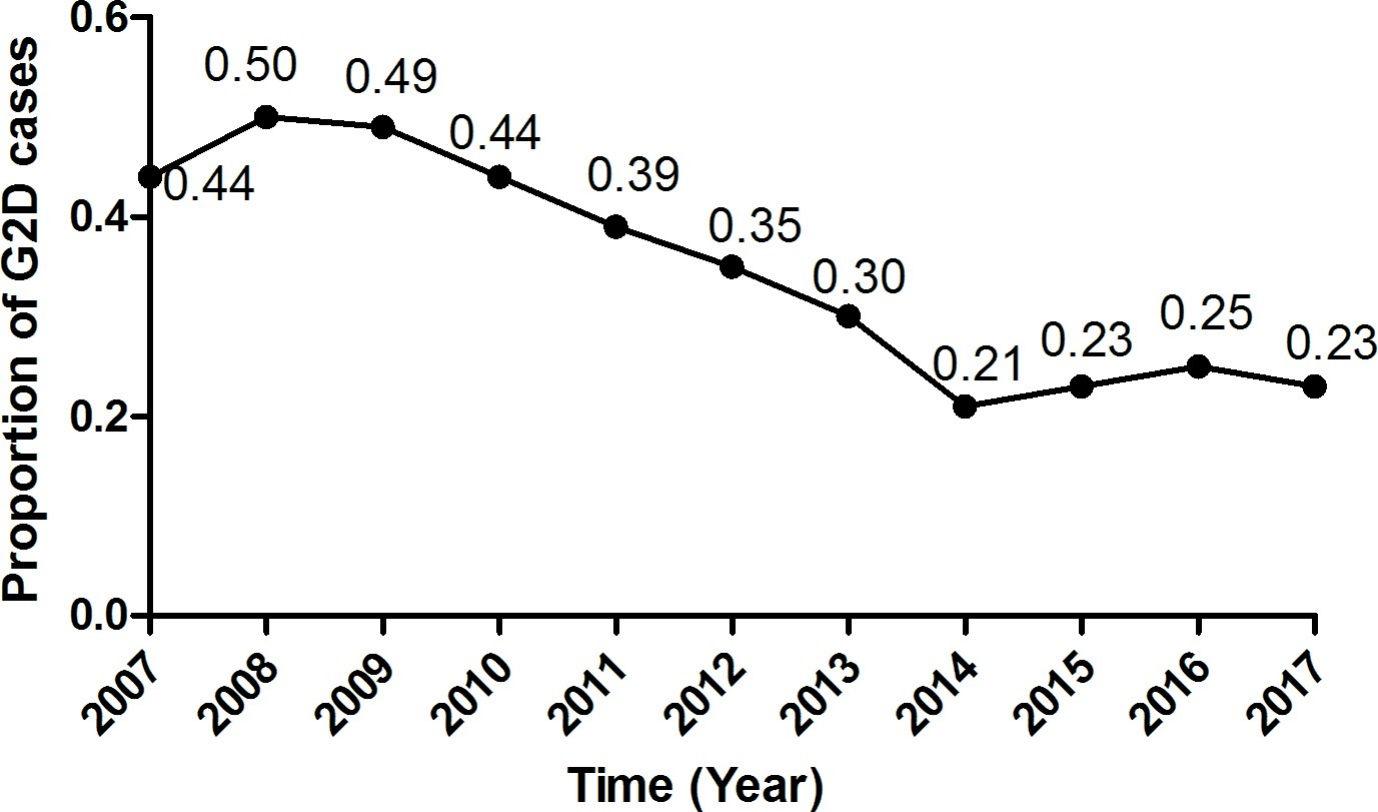
The proportion of G2D of newly detected leprosy cases over the years.

92 (39.8%) cases presented with grade 2 disability at diagnosis. Fig 2 shows the annual proportion of G2D among new cases. The annual G2D proportion showed an obvious declining trend between 2007 and 2017, from 50% in 2008 to 23% in 2017.

The mean patient delay, health service delay and total delay of G2D cases were all significantly longer than that of cases without G2D. Of the 92 cases with G2D, the proportions of cases with patient delay of >12 months (44/90, 48.9%), total delay of >24 months (75/92, 81.5%), PB type (28/92. 30.4%) and known infectious source (36/92, 39.1%) were significantly higher than that of cases without G2D. Nearly two thirds (61/92, 66.3%) of the G2D cases were detected by skin clinics and this proportion is significantly lower than that of cases without visible disabilities (120/139, 86.3%). The association between G2D and demographic and disease factors is shown in Table 1. The ratio of male, illiterate among G2D cases was slightly higher than that of non-G2D cases, but the difference didnot reach a significant level.

**Table 1:** Associated factors of G2Damong newly detected leprosy cases during2007-2017.

Variable	Disabilitystatus		OR (95%CI)	P value
	Non-G2D cases	G2D cases		
	N(%)	n(%)		
**Sex**				
Male	76**(**54.7)	54**(**58.7)	1	P = 0.547
Female	63**(**45.3)	38**(**41.3)	0.85 (0.50–1.45)	
**Age(years)**	50.6±16.6	53.4±15.0		P = 0.173
≦35 >35	22**(**15.8)	6**(**6.5)	1	P = 0.072
	117**(**84.2)	86**(**93.5)	1.88 (0.94–3.72)	
**Education level**				
Illiterate	40**(**28.8)	33**(**35.9)	1	P = 0.257
Literate	99**(**71.2)	59**(**64.1)	0.72 (0.41–1.27)	
Patient delay in months ($\bar{x}$ ±SD)	19.3±42.6	46.4±75.5		P = 0.002
≦12	98(71.5)	46(51.1)	1	P = 0.002
>12	39(28.5)	44(48.9)	2.404(1.38–4.19)	
Missing	2	2		
Total delay in months ($\bar{x}$ ±SD)	47.1±56.7	89.8±83.8		P<0.001
≦24	69(49.6)	17(18.5)	1	P<0.001
>24	70(50.4)	75(81.5)	4.35 (2.33–8.11)	
**Classification**				P = 0.002
Pauci-bacillary	19(13.7)	28(30.4)	2.76(1.43–5.32)	
Multi-bacillary	120(86.3)	64(69.6)	1	
**Detection mode**				
Skin clinic Others	120(86.3)	61(66.3)	1	P<0.001
	19(13.7)	31(33.7)	3.21 (1.68–6.14)	
**Infectious source**				
Known	37(26.6)	36(39.1)	1.77 (1.01–3.12)	P = 0.046
Unknown	102(73.4)	56(60.9)	1	

## Discussion

The sharp decrease of reported leprosy prevalence once raised a sense of alarm among authors. In the past two decades, active case-finding activities in some countries had verified that there were a large number of undetected cases in the community [19], which revealed the real burden of leprosy. Furthermore, with more than 210,000 new cases were detected annually, leprosy is far from eradicated [20]. Even in post-elimination era, efforts are still needed to further reduce the disease burden and to sustain necessary control activities.

Nowadays, early detection and complete treatment with MDT remain the fundamental principles of leprosy control [15]. How to pick up the few scattered leprosy cases latent in a large population poses a severe challenge for China and the entire world as well. Early diagnosis of leprosy is not easy. With insidious onset, chronic progression and asymptomatic skin lesions, leprosy is often ignored by sufferers [11]. With variable clinical manifestations, ranging from single hypo-pigmented maculae to widespread nodules, plaques and infiltrations along with generalized visible deformities or disfigurations [12], leprosy can mimic many other skin diseases and neurological conditions, making early diagnosis difficult.

Evidence has demonstrated that disability-based targets encourage early diagnosis [9]. To some extent, G2D reflects the level of awareness of early signs and symptoms of leprosy in the public and the capacity of health system to recognize and treat leprosy at an early stage [15]. Compared with other countries, the proportion of cases with G2D among newly detected cases had been high in China [21]. Compared with endemic provinces, such as Sichuan and Yunnan, low-endemic provinces including Shandong had an even higher G2D proportion. In 2008, this proportion in Shandong province hit 50%, which indicated that it was essential to strengthen case-finding activities.

Early detection is known to be the most important factor in preventing disability from leprosy [22]. The past ten years of practice showed that comprehensive measures in Shandong province succeeded in reducing the proportion of G2D among newly detected leprosy cases from50% in 2008 to 23% in 2017. As for Shandong, although the rate of newly detected cases with G2D has already been reduced to less than 1 per 100,000, with a G2D proportion of more than 20%, there is still room for improvement in early detection. In addition, most of the newly detected cases were MB patients, which also indicated the presence of advanced cases of leprosy [15] and indirectly, delay in diagnosis.

Innovative and active case finding methods was one of the identified key processes in implementing the global strategy [15]. Monitoring of leprosy symptoms, a new kind of active case-finding activity, changed skin-clinics from passive case-finding to active surveillance, and from temporary intensified campaigns to routine monitoring. 13 new cases are detected in 2017, the first year of symptom surveillance, showing an obvious increase from 8 cases in 2016. Except for one self-reported case, all the other 12 cases were detected through symptom surveillance. This method may play an important role in early detection of leprosy in the future, especially in post-elimination era and in low endemic areas.

To further reduce the proportion of G2D among new cases, it is also essential to understand the barriers to early detection [15]. In our study, we found that cases with known infectious source had higher G2D proportion than those without, which may suggest the existence of stigma in the community. This finding supports research in Brazil, which found that cases were more likely to wait longer before presentation for fear of community isolation [10]. Health promotion activities had been successful in increasing knowledge about leprosy [16]. To shorten patient delay, it is necessary to develop specific and targeted health education activities for the general population, so as to remove wrong ideas and prejudices among the public against leprosy and promote self-reporting and early presentation.

This study reaffirmed that longer delay in diagnosis is associated with G2D. To shorten the health service delay, better training of personnel at grassroots levels and dermatological clinics, with the purpose of maintaining a high suspicion, is recommended [11]. We found that among non-G2D cases, the proportion of cases detected by skin clinics was significantly higher than that of cases with G2D. This indicated that dermatologists have better knowledge and skill in early diagnosis of leprosy. Leprosy control program manager should further strengthen the relationship with dermatologists, to encourage skin clinics at different levels to play more important role in early detection of leprosy cases [23]. To encourage report of suspected cases of leprosy, some kind of incentive measures also can be used, given that leprosy has become a rare disease in most parts of the world and awareness of leprosy was low among medical staff.

With the development of society and economy, the mobility of population, including leprosy cases, is also increasing. New cases originated from transient or mobile population is another issue of great concern, because they are more difficult to detect and this will increase the risk of developing G2D. In the last decade, it was reported that among the newly detected cases, 37% in Saudis Arabia [24], 73.9% in Kuwait [25], nearly 60% in UK [26] were foreign born cases. In 2015, the proportion of foreign born individuals among newly detected cases were more than 40% in Malaysia and nearly 25% in Thailand [6]. In China, 4775 new cases of leprosy were reported during 2011–2015, including 518 (10.8%) patients detected in floating people [27]. Our study found that more than one sixth of the new cases generated from immigrated population, most of whom were full-time housewives or unskillful laborers coming from leprosy endemic provinces. How to improve early detection of cases latent in mobile population deserves further exploration.

One of the limitations of this study is the accuracy of recall. Since leprosy is a chronic disease, it is difficult for the cases, especially for those with long diagnosis delays, to remember the exact time of disease onset, help-seeking activities, etc. The other thing is that this study was limited in Shandong province, perhaps can represent some coastal provinces in China, but may not be applied to all parts of the country, due to differences in leprosy control phases, government commitments and uneven distribution of health resources, such as skin clinics and dermatologists, in different provinces or areas.

### Conclusion

Steady decline of prevalence rate, lack of cases among children, late onset and high MB proportion all indicate that leprosy perhaps is dying out in Shandong province. However, a few scattered leprosy cases coming mainly from former high or middle endemic areas, some with long delay in diagnosis and visible disabilities, still can be seen. The latent cases among immigrants make early detection of cases even more difficult. To promote early diagnosis of leprosy in post-elimination era, government commitment is vital to provide financial security and policy support. Comprehensive measures including health promotion, personnel training, reward-offering and symptom surveillance are also necessary to improve early presentation of individuals and to increase suspicion and encourage participation of all relevant medical staff. A powerful referral center is also essential to establish or exclude the diagnosis of leprosy accurately.

## Supporting information

S1 Checklist STROBE checklist. (DOC)
