# Supplementary material for: Comprehensive measures succeeded in improving early detection of leprosy cases in post-elimination era: Experience from Shandong province, China
Source: PLoS Negl Trop Dis. 2020 Feb 20;14(2):e0007891. doi: 10.1371/journal.pntd.0007891 (PMC7062278; doi:10.1371/journal.pntd.0007891)
Supplement: S1 Checklist — (DOC) [file pntd.0007891.s001.doc]

STROBE Statement—Checklist of items that should be included in reports of ***cross-sectional studies***

|  | Item No | Recommendation | section | Paragraph |
| --- | --- | --- | --- | --- |
| **Title and abstract** | 1 | (*a*) Indicate the study’s design with a commonly used term in the title or the abstract | abstract | 1-2 |
|  |  | (*b*) Provide in the abstract an informative and balanced summary of what was done and what was found | abstract | 2-4 |
| Introduction | | |  |  |
| Background/rationale | 2 | Explain the scientific background and rationale for the investigation being reported | Introduction | 1-6 |
| Objectives | 3 | State specific objectives, including any prespecified hypotheses | Introduction | 6 |
| Methods | | |  |  |
| Study design | 4 | Present key elements of study design early in the paper | Materials & Methods | 2 |
| Setting | 5 | Describe the setting, locations, and relevant dates, including periods of recruitment, exposure, follow-up, and data collection | Materials & Methods  Comprehensive measures adopted | 2-3  1-7 |
| Participants | 6 | (*a*) Give the eligibility criteria, and the sources and methods of selection of participants | Materials & Methods | 1 |
| Variables | 7 | Clearly define all outcomes, exposures, predictors, potential confounders, and effect modifiers. Give diagnostic criteria, if applicable | Materials & Methods | 1 |
| Data sources/ measurement | 8* | For each variable of interest, give sources of data and details of methods of assessment (measurement). Describe comparability of assessment methods if there is more than one group | Materials & Methods | 1,3 |
| Bias | 9 |  |  | NR |
| Study size | 10 | Explain how the study size was arrived at |  | NR |
| Quantitative variables | 11 | Explain how quantitative variables were handled in the analyses. If applicable, describe which groupings were chosen and why | Materials & Methods | 3 |
| Statistical methods | 12 | (*a*) Describe all statistical methods, including those used to control for confounding | Materials & Methods | 3 |
|  |  | (*b*) Describe any methods used to examine subgroups and interactions |  | NR |
|  |  | (*c*) Explain how missing data were addressed | Materials & Methods | Table 1 |
|  |  | (*d*) If applicable, describe analytical methods taking account of sampling strategy |  | NR |
|  |  | (*e*) Describe any sensitivity analyses |  | NR |
| Results | | |  |  |
| Participants | 13* | (a) Report numbers of individuals at each stage of study—eg numbers potentially eligible, examined for eligibility, confirmed eligible, included in the study, completing follow-up, and analysed | Results | 1 |
|  |  | (b) Give reasons for non-participation at each stage | Results | 1 |
|  |  | (c) Consider use of a flow diagram |  | NR |
| Descriptive data | 14* | (a) Give characteristics of study participants (eg demographic, clinical, social) and information on exposures and potential confounders | Results | 1,2 |
|  |  | (b) Indicate number of participants with missing data for each variable of interest |  | Table 1 |
| Outcome data | 15* | Report numbers of outcome events or summary measures | Results | 3 |
| Main results | 16 | (*a*) Give unadjusted estimates and, if applicable, confounder-adjusted estimates and their precision (eg, 95% confidence interval). Make clear which confounders were adjusted for and why they were included |  | Table 1 |
|  |  | (*b*) Report category boundaries when continuous variables were categorized |  | Table 1 |
|  |  | (*c*) If relevant, consider translating estimates of relative risk into absolute risk for a meaningful time period |  | NR |
| Other analyses | 17 | Report other analyses done—eg analyses of subgroups and interactions, and sensitivity analyses |  | NR |
| Discussion | | |  |  |
| Key results | 18 | Summarise key results with reference to study objectives |  | 3,4,6-8 |
| Limitations | 19 | Discuss limitations of the study, taking into account sources of potential bias or imprecision. Discuss both direction and magnitude of any potential bias |  | 9 |
| Interpretation | 20 | Give a cautious overall interpretation of results considering objectives, limitations, multiplicity of analyses, results from similar studies, and other relevant evidence |  | 10 |
| Generalisability | 21 | Discuss the generalisability (external validity) of the study results |  | 9 |
| Other information | | |  |  |
| Funding | 22 | Give the source of funding and the role of the funders for the present study and, if applicable, for the original study on which the present article is based |  | Funding |
